# Macaulay on Small-Pox

**Published:** 1888-11-03

**Authors:** 


### MACAULAY ON SMALL-POX.

In The Hospital of last Saturday occurs a quotation from Macaulay, on the subject of small-pox and vaccination, that I seem to have read before. It is the rather sensational paragrap habout the "hideous disease" that "filled the churchyards with corpses, and turned the babe into a changeling at which its mother shuddered, and made the eyes and cheeks of the betrothed maiden objects of horror to the lover." It was curious that Macaulay forgot to state that small-pox, then as now, was a singularly varying disease, and that, i often fatal, it was far oftener so extremely slight that the patient "scarcely ailed at all," as a physician of the eighteenth century wrote of it. But still more curious was it, that Macaulay did not appear to see that the conditions as to sanitation, under which the people lived in the seventeenth and eighteenth centuries, were quite sufficient to account for the prevalence of small-pox as of other diseases now unknown, banished altogether by the sanitary measures which, whenever they are carried out, are found sufficient to banish small-pox without any aid from vaccination. On the same page of The Hospital that gives this passage from Macaulay is a paragraph to the effect that "the history of the epidemics of the middle ages is the most fearful and murderous that can be imagined," and it adds that the "Black Death" killed in London 100,000, and in Norwich alone 50,000." But where is the Black Death now? Has it not for ever vanished with the fearful state of things that made its ravages possible? And where also is the logic of attributing solely to vaccination our lessened small-pox, while admitting that to sanitation we owe our freedom from the Black Death, the Plague, the "Sweating Sickness," and many other equally fatal diseases.—S. W., Edgbaston, Sept. 25th.

